# Facilitators and barriers to home-based toothbrushing practices by parents of young children to reduce tooth decay: a systematic review

**DOI:** 10.1007/s00784-021-03890-z

**Published:** 2021-03-20

**Authors:** Elnaz Aliakbari, Kara A. Gray-Burrows, Karen A. Vinall-Collier, Sakina Edwebi, Ama Salaudeen, Zoe Marshman, Rosemary R. C. McEachan, Peter F. Day

**Affiliations:** 1 Clarendon Dental Spa, Leeds, UK; 2 School of Dentistry, Faculty of Medicine and Health, University of Leeds, Leeds, LS2 9JT UK; 3 School of Clinical Dentistry, Faculty of Medicine, Dentistry & Health, University of Sheffield, Sheffield, UK; 4 Bradford Institute for Health Research, Bradford, UK; 5 Bradford Community Dental Service, Bradford District Care NHS Foundation Trust, Bradford, UK

**Keywords:** Oral health, Barriers, Facilitators, Children, Parents, Theoretical Domains Framework

## Abstract

**Objectives:**

Parental supervised toothbrushing (PSB) is a collection of behaviours recommended by national guidance to improve oral health. This systematic review aimed to identify the barriers and facilitators to PSB.

**Materials and methods:**

Studies investigating parental involvement in home-based toothbrushing in children under 8 years old and the impact on tooth decay were included. Electronic databases, references and unpublished literature databases were searched. The Theoretical Domains Framework (TDF) was used to code barriers/facilitators to PSB.

**Results:**

Of the 10,176 articles retrieved, 68 articles were included. Barriers and facilitators were found across all 12 TDF domains. Barriers included an inadequate toothbrushing environment and resources, knowledge of what PSB entails and child behaviour management. Facilitators were increased oral health knowledge, the adaption of the social environment to facilitate PSB and positive attitudes towards oral health. When only high-quality articles were synthesized, knowledge was not a common barrier/facilitator.

**Conclusions:**

There are a comprehensive range of barriers/facilitators to PSB acting across all domains and at multiple levels of influence. This review identifies the most popular domains, thus informing the focus for supporting resources to supplement oral health conversations.

**Clinical relevance:**

PSB is a complex behaviour. Practitioners need to understand and be able to explore the wide range of potential barriers and have practical suggestions to enable PSB. This review provides pragmatic examples of different barriers and facilitators and emphasises the importance of listening to parents and exploring their story to identify the barriers and solutions that are relevant to each family.

**Supplementary Information:**

The online version contains supplementary material available at 10.1007/s00784-021-03890-z.

## Background

Toothbrushing with fluoride toothpaste is an apparently simple yet effective behaviour for preventing tooth decay (caries) [1]. Paradoxically, although preventable, tooth decay is the most prevalent condition in children and remains a key international public health priority [2]. Furthermore, tooth decay is a disease of health inequality. For example, in some parts of the UK, typically the most deprived areas, just under half of children have tooth decay affecting multiple teeth by the age of 5 [3]; and it is the most common reason why young children have a general anaesthetic [4, 5].

UK and other national guidance recommend a collection of toothbrushing behaviours for young children and for this review they have been summarised under the term parental supervised toothbrushing (PSB). PSB includes twice daily brushing under supervision using an appropriate amount and strength of fluoride toothpaste from the emergence of the first tooth up to at least 7 years old [6–8]. PSB is a dyadic process [9], which entails parents actively brushing their children’s teeth and children allowing their teeth to be brushed; as such, it is a complex behaviour with many influences at individual (parent and child separately), interpersonal (parent and child interactions) and wider societal and environmental levels. Furthermore, PSB is composed of a collection of behaviours beyond oral health practices, such as parenting; and due to the various socio-ecological influences (for example, the cost and accessibility of dental resources, and the influence of family and friends) on PSB, it can be a difficult behaviour to perform [10]. Establishing effective oral health habits in early life is a key indicator of long-term oral health [11–13].

Although it appears initially that PSB is a simple set of behaviours, it is in fact a complex behaviour due to the interplay between different behaviours, individuals and the influence of the environment. As a complex behaviour, development of interventions to address it or the evaluation of such interventions requires a suitable methodology. The Medical Research Council (MRC) provides detailed guidance on how to apply such methods in the development and evaluation of complex interventions and highlights the importance of comprehensively understanding the problem and context in the initial stages of intervention development. Thus, to effectively promote PSB, it is important to understand the barriers and facilitators which affect performance of this behaviour. Barriers refer to any contributing factors or behavioural determinants which prevent PSB from taking place, whereas facilitators are factors or determinants (including the reversal of barriers) that promote PSB. While several studies have investigated barriers and facilitators of PSB, these have not been summarised in a systematic manner with reference to behaviour change theory. Therefore, the aims of this systematic review were (1) to identify the barriers and facilitators to PSB and map them in a meaningful way using behaviour change theory, and (2) to identify associations between barriers and facilitators and parental supervised toothbrushing and tooth decay.

## Methods

### Search and inclusion/exclusion criteria

Literature searches were undertaken up to May 2016 by an information specialist on a number of databases, including MEDLINE, EMBASE, PubMed, Web of Science, PsycINFO, Scopus and the Cochrane Library using the search terms ‘toothbrushing’, ‘tooth decay’, ‘children’ and ‘parent/carer’. References of included studies and ‘near misses’ were checked to identify other relevant publications and unpublished literature was electronically searched through ClinicalTrials.gov and the National Research Register. The search strategy and full protocol were registered on the PROSPERO website [14] and the search strategy is provided in the Supplementary Materials. These searches were updated in November 2019 to include any research published since the original literature searches were conducted following the same previous search strategy.

The title and abstract of the identified articles were evaluated by three researchers (EA, SE, KG-B) for whether they met the inclusion criteria. The full texts were independently reviewed by four reviewers (SE, KG-B, EA, AA) for inclusion/exclusion and the reason for exclusion was recorded. KG-B provided oversight with support from the remaining authors (PD, ZM, RM) over searching, identification, selection and data extraction.

Studies were included if they investigated parental involvement in toothbrushing in children under 8 years old and available in English. Studies were excluded if (i) there was no parental involvement; (ii) they examined school- or nursery-based toothbrushing; (iii) they included children 8 years old and above where it was not possible to identify the data specifically relating to the children under 8 years old; (iv) they investigated the effectiveness of toothbrushing on plaque removal or improving gingival health; (v) they did not report primary data (e.g. editorials, commentaries, discussion pieces); and (vi) they investigated children with disabilities (including learning, physical and medical) where these disabilities may necessitate children requiring long-term parental toothbrushing.

### Coding

Following a preliminary screening of abstracts and titles, the abstracts of 10% of the potentially relevant studies were screened by all the authors against the inclusion/exclusion criteria and any disagreement was discussed and a consensus agreed. Five reviewers (EA, KV-C, KG-B, SE, AA) screened the remainder of titles and abstracts independently to identify potentially relevant studies. For those studies which met, or appeared to meet the inclusion criteria, the full text of the study was reviewed by thereviewers independently. Full papers that did not meet the inclusion criteria at this stage were excluded and the reasons for exclusion recorded. References in the identified studies were checked and other studies were included where relevant, and duplicates were recorded and discarded.

For studies meeting the inclusion criteria, data extraction was undertaken using the customised data extraction pro forma for included studies by five reviewers independently. This data extraction process was piloted by the authors to ensure the approach was appropriate and enabled collection of the relevant data by each member extracting data from several papers each and discussing their findings. From this process, a consensus was reached, and the data extraction form finalised. Once this process was completed, the reviewers met and examined if similar data had been extracted from each included paper. Discrepancies were resolved by consensus or recourse to an additional researcher where necessary.

### Theoretical Domains Framework (TDF)

The Theoretical Domains Framework (TDF) [15] was used as a tool to enable a systematic approach to data synthesis. The TDF is a psychological framework that outlines 12 key domains that explain health behaviour, which have been derived from 33 behaviour change theories. In the current review, the TDF was adapted to reflect toothbrushing behaviours. Table 1 provides a list of the 12 domains and gives examples of how different PSB barriers would be categorised. Each paper was assessed for any description of a barrier or facilitator to PSB, and this data extracted verbatim. Each description was then coded in conjunction with our adapted TDF to ascertain which of the domains most accurately reflected the description of the barrier and/or facilitator in the relevant papers by a behavioural scientist (KG-B) along with three researchers (EA, SE, AA). Each description and accompanying coding were discussed by the reviewers to ensure agreement.

**Table 1:** Distribution of the number of times studies identified constructs of the Theoretical Domains Framework (TDF) as barriers and/or facilitators for the whole data set and for the top third of highest quality papers (top three highlighted within each category)

		Whole data set (*n* = 68)		Highest quality papers (*n* = 8)		Significant association with oral health outcomes
TDF construct	Example	Number of times identified as a barrier	Number of times identified as a facilitator	Number of times identified as a barrier	Number of times identified as a facilitator	
Knowledge	Knowledge around toothbrushing (introduction, timing, frequency, toothpaste, rinsing, how to brush children’s teeth, supervised toothbrushing recommendation)	24	30	1	1	
Social influences	Social support (family, health professionals, school, etc.)	18	29	3	4	
Environmental context and resources	Competing demands on time	22	3	3	0	
Beliefs about consequences	Attitudes/beliefs about toothbrushing	13	19	1	2	
Behaviour regulation	Child’s behaviour (compliance/resistance)	20	9	5	3	X
Beliefs about capabilities	Perceived competence to brush teeth	16	16	3	1	
Skills	Parent’s skills around toothbrushing	12	11	3	3	
Nature of behaviour	Toothbrushing routines	4	9	3	2	
Motivation and goals	Toothbrushing as a goal priority	6	8	1	1	
Emotion	Fear of dental treatment	4	5	1	2	
Social/professional role and identity	Perception of own role in children’s dental care	1	3	1	1	
Memory, attention and decision processes	Remembering to brush children’s teeth	2	1	1	0	

Barriers and facilitators were not mutually exclusive to papers and both categories could appear multiple times within a paper

### Associations between barriers/facilitators and oral health outcomes

For each included study, it was assessed if the authors had reported any associations between the barriers and facilitators with PSB behaviour and/or tooth decay. Such associations are reported in the Supplementary Materials^1^. Due to the wide range of studies included in the present review, outcomes and measures were necessarily assessed narratively.

### Quality assessment

The quality assessment tool (QATSDD), developed by Sirriyeh, Lawton [16], was used to assess the quality of all included studies. This tool includes 16 items, scored between 0 and 3, and can be applied to studies using different methodological approaches (e.g. quantitative, qualitative and mixed methods). Applying this tool, each paper was given a quality score ranging between 0 and 48, and the sum of these provided an overall score for the body of evidence. This was undertaken independently by four reviewers (EA, KG-B, SE, AA) and disagreements were resolved by discussion. Due to the large variance in study quality and the impact methodological quality can have on subsequent results, it was decided to synthesise the results in two ways to ensure we were obtaining the most comprehensive and pertinent results. As such, initially all the included study findings were synthesised regardless of quality score. Following this, the studies were categorised as good, fair or poor (Helfand and Balshem, 2009) and a subgroup of the highest scoring papers (i.e. the top third scoring 32 and above) were synthesised to explore whether there was a difference in the barriers and facilitators identified in the highest quality papers compared to the whole sample of papers.

## Results

Due to the extensive nature of the present review, the results are discussed in the following order: study characteristics; quality assessment (including subgroup synthesis of the highest quality scoring studies); mapping barriers and facilitators onto the TDF (for whole review sample) and associations between barriers/facilitators and oral health outcomes.

### Study characteristics

**Fig. 1:**
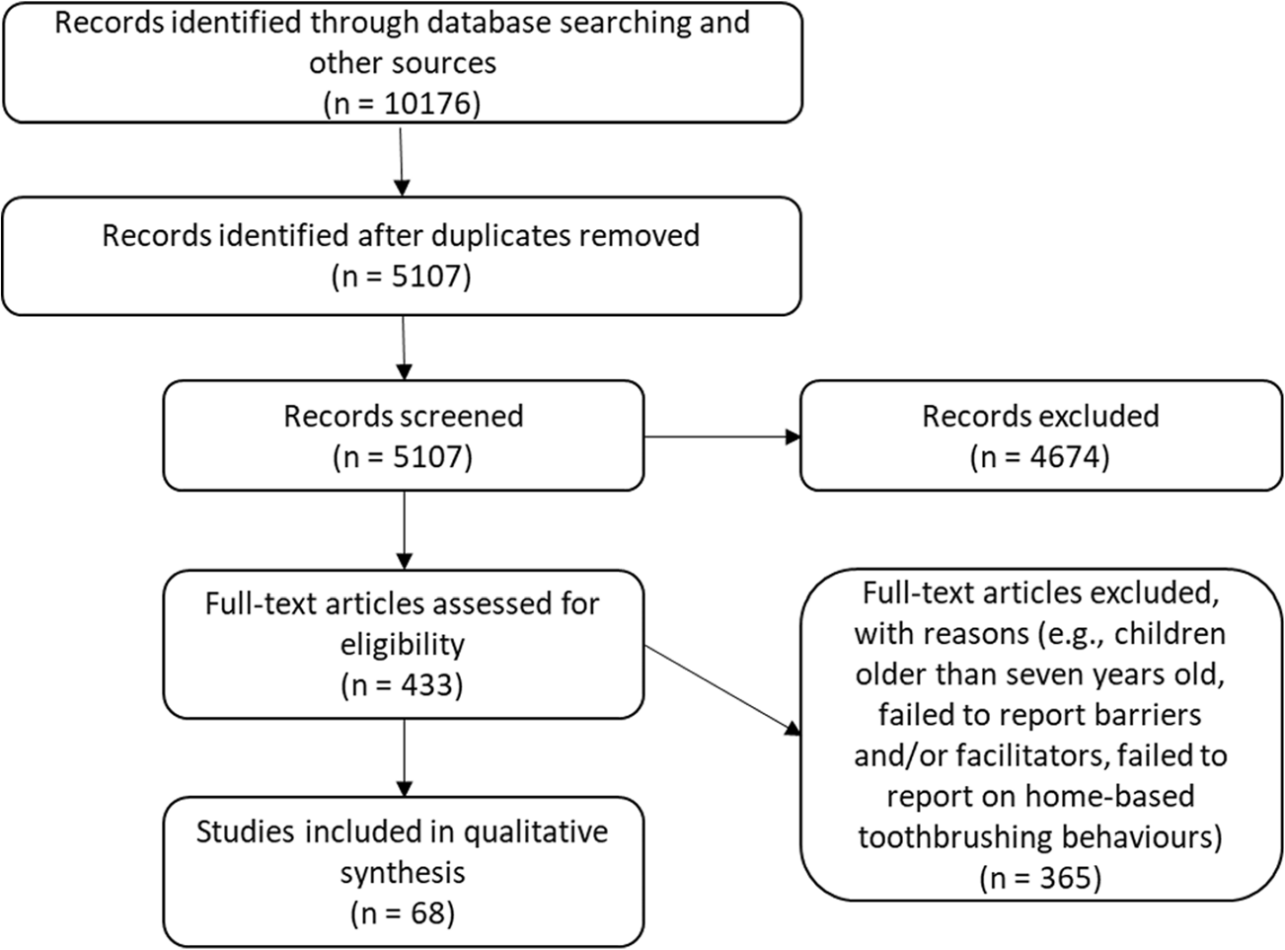
Systematic review search strategy and screening process

Initial screening identified 5107 papers eligible for inclusion after duplicates removed, 433 underwent full-text analysis, and 68 studies between 1978 and 2019 were identified as meeting the inclusion criteria and data extracted (Fig. 1). The summary of studies investigating barriers and facilitators to PSB are reported in the Supplementary Materials and the full reference list of included studies can be found in Supplementary Materials 2. Fifty-six provided quantitative data, nine provided qualitative data, and three were mixed methods. Studies were undertaken worldwide. Sample sizes ranged from 15 to 9722 participants with participants from a range of different ethnic groups. Parents/caregivers’ ages ranged between 15 and 70 years, and childrens ages ranged from 0 to 7 years old. Barriers and facilitators were identified in the studies in a variety of ways, including from predefined questionnaires, qualitative interviews and suggestions from the author within the paper. Descriptions of barriers and facilitators identified from the papers along with their TDF coding are outlined in the Supplementary Materials.

### Quality assessment

The quality scores for studies included within the review ranged from 8 to 39 (median 19, IQR 16–23) and are reported in the Supplementary Materials. Most of the studies were proficient in detailing their aims and objectives, research setting, data collection procedure and recruitment data. They had reasonably sized and representative samples and a good fit between the research question and method of analysis. However, the rationale for data collection tools and justification for analytical method were less well described. There was also little use of theoretical frameworks and user involvement in the planning of the study design via the use of pilot studies or consultation with stakeholders/general population. For the quantitative studies, the fit between the research question and method of data collection was good, but there was a lack of assessment of the reliability and validity of measurement tools. The assessment of reliability (e.g. triangulation, coding by multiple researchers) was equally poor in qualitative studies (see quality assessment scores in the Supplementary Materials).

The highest quality papers ranged in score from 32 to 39 and differed from the rest of the included papers in both design and frequency of barriers and facilitators. Most of the high-quality papers included a qualitative component (*n* = 3), with remaining studies using either an observational (*n* = 1) or quantitative (questionnaire, *n* = 1) design. Most notable, however, was the difference between the most common barriers and facilitators identified by these high-quality studies. Although, yet again, all 12 TDF domains were identified, in terms of barriers, behaviour regulation and environmental context and resources remained key domains; social influences, belief in capabilities, nature of behaviour and skills also featured as key barriers to PSB. In terms of facilitators, social influences remained a key domain, but behaviour regulation and skills emerged as key facilitators to PSB. Within these studies, knowledge no longer featured in the top three of barriers or facilitators and beliefs on consequences was no longer within the top three facilitators (see Table 1 for a comparison of the frequencies across the whole sample and the top ten highest quality papers).

### Mapping barriers and facilitators onto the TDF

Following the independent mapping of the identified barriers and facilitators onto the constructs defined by the TDF by four coders, it was found that all 12 defined constructs were evident in the literature. It is important to acknowledge that domains were not treated as mutually exclusive; thus, where a barrier or facilitator was deemed to cover several domains, it was coded as such (see the Supplementary Materials for each barrier/facilitator and its TDF coding).

With regard to barriers to PSB, all 12 domains were identified as influential in preventing PSB from taking place (Table 1). The most common barriers identified were knowledge, environmental context and resources, and behaviour regulation.

- The problems with knowledge were generally twofold. First, there was a lack of knowledge about the importance of primary teeth [17–20]. Second, there was a lack of knowledge about toothbrushing [21], including when to start brushing a child’s teeth [22], whether a child needed assistance brushing [23] and how to brush young children’s teeth [24] (e.g. positioning [25], frequency [26], what toothpaste and amount to use [25, 27], rinsing after brushing [27], plus a general lack of knowledge about fluoride and how to identify fluoride content [25, 28]).
- Barriers in relation to the environmental context and resources were related to the lack of access and cost of dental services, dental provisions and oral health information [26, 28–32]. Furthermore, parents have busy schedules, and with conflicting demands placed upon them, they lack time and availability to actively brush their child’s teeth [20, 24, 31, 33–36]. The night-time brush is made particularly difficult when competing with the tiredness of the child [20, 37].
- Regarding behaviour regulation, the barriers related both to the child’s temperament [36, 38] and behaviour [33] and how the family functioned to manage their child’s behaviour [39]. Difficulty supervising/assisting toothbrushing was found when children were resistant to having their teeth brushed [26]. This resistance could manifest in two distinct ways: the first being a child who was uncooperative and non-compliant with toothbrushing, thus actively refusing and avoiding toothbrushing [20, 24, 31, 35, 37, 40–44]. In contrast, the second way was resistance specifically to parental involvement in toothbrushing, with children wanting to exert their own independence [45], particularly with increasing age [46]. Furthermore, in some instances, such independence was encouraged by parents [28]. Indeed, how parents managed their children’s behaviour while toothbrushing was a key barrier, with ineffective parenting strategies leading to poorer toothbrushing outcomes [37, 41, 43].

With regard to facilitators to PSB, all 12 domains were identified as influential in enabling PSB to take place (Table 1). The most common facilitators reported were knowledge, social influences and beliefs about consequences.

- With regard to knowledge, parents having good knowledge about oral health [20, 23, 36, 37, 47–49], including the causes and consequences of poor oral health [21, 50, 51], and knowing  about the preventative role of toothbrushing and fluoride was conducive to PSB [22, 27, 52]. This was perceived to be further facilitated through the early provision of oral health education [17, 31, 53–55].
- With regard to social influences, parents with good oral health practices, including regular dental attendance, provide a family norm of good oral health care and can serve as a role model for their children [19, 21, 34, 35, 54, 56–60]. Where the people around them also have positive attitudes, and can provide learning and support, this also provides a social norm of good oral health [31, 34, 35, 37, 61–63]. Finally, the support of the community, schools and empathetic health professionals, including dentists, general practitioners and paediatricians, is conducive to good oral health behaviour [17, 20, 33, 37, 45, 51, 55, 56].
- Regarding beliefs about consequences, having generally positive attitudes about oral health [36, 51] as well as positive attitudes towards the importance of toothbrushing, helping children to brush and the ability to brush children’s teeth were facilitators to PSB [34, 35, 54, 58, 63–67]. In addition, understanding the consequences of poor oral health [21, 37] and the benefits of adhering to oral health recommendations, such as toothbrushing (e.g. better sleep, appearance) [26, 49, 62], was faciliatory, with this information being gained through dental visits [48] and based on parents’ own positive and negative experiences of oral health [36].

### Associations between barriers/facilitators and oral health outcomes

Forty-two studies included in the review explored whether there were significant relationships between the various demographic factors, barriers and facilitators, toothbrushing behaviour and tooth decay development (see the Supplementary Materials). These were naturally occurring relationships and therefore not the result of any experimental manipulation or intervention and will now be discussed in turn.

### Parental supervised toothbrushing behaviour

Significant associations were reported between PSB and knowledge [23, 48], motivation and intentions [48, 63, 68], parental habits [59], attitudes [63], beliefs (evaluative and behavioural), perceived role of the child and partner [69], parental confidence (self-efficacy) [48, 68, 70], child’s temperament [38], family functioning [39], parents’ dental attendance [56] and social norms [63]. Furthermore, in an observational study of PSB, Zeedyk [42] found that parents’ perceptions ofPSB did not align with their behaviours shown during a self-filmed PSB session.

With regard to the perceived importance of good oral health behaviours for children, significant associations were reported between parental attitudes towards their child’s and own oral health and behaviour and understanding the importance of children developing oral hygiene skills [58]. Another study identified a lack of knowledge and awareness of the importance of primary teeth as significant barriers to preventative dental care [18].

### Tooth decay

Similarly, many of the barriers and facilitators to PSB were found in the literature to have significant associations with the development of tooth decay. Significant associations were also found between tooth decay and attitudes towards toothbrushing [65, 71], onset of toothbrushing [72], toothbrushing frequency [60], duration of toothbrushing [41], parental supervised/assisted toothbrushing [40, 41, 60], toothbrushing efficacy [65], perceived ability to incorporate regular toothbrushing into a child’s routine [67], the child’s temperament [44], parents’ own toothbrushing practices [71], parenting skills [41, 43] and knowledge [47]. However, one study did not find a significant association between PSB and tooth decay [63], and a further three studies failed to find a significant association between tooth decay and knowledge, attitudes toward dental care, child temperament and dental-seeking behaviour [38, 55, 60]. Generally, the studies that reported associations between barriers/facilitators, PSB behaviours and/or tooth decay tended to support the assumption that barriers lead to greater levels of tooth decay and facilitators reduced tooth decay prevalence through their impact on PSB behaviours. Although, many of these associations were attenuated by demographic factors. Furthermore, the reported associations did not always find such positive relationships and some of the study designs used did not allow causation associations to be examined.

### Demographic factors

In addition, various socio-demographic factors were found to be associated with the prevalence of oral health attitudes, behaviour and tooth decay development or attenuated these relationships. These factors included socioeconomic status [43, 56, 61, 64, 72], ethnicity [46, 48, 50, 69, 71, 73–75], language [54], educational level [22, 47–49, 53, 61, 65, 71, 72, 76], parental age [48, 68, 72], child’s age [46], transience (e.g. migration) [20, 46, 49, 50, 73], location (i.e. urban vs rural) [72], family size [22, 60, 72] and birth order [72].

## Discussion

This is the first systematic review to synthesise the research examining the barriers and facilitators to home-based toothbrushing practices used by parents. A total of 68 studies were included in the review addressing the key objective of identifying the barriers and facilitators to PSB.

A wide variety of barriers and facilitators were identified in the literature. Knowledge was identified most frequently in the literature as both a barrier and facilitator (i.e. lack of knowledge vs good knowledge about oral health). However, many studies were limited in the range of barriers and facilitators studied due to the measures they employed, which means that this may be an artefact of the studies included in the current review. In fact, a recent qualitative study highlighted that knowledge was not the key driver of behaviour and that barriers related more to ‘how’ to perform oral health care rather than ‘what’ to do [37]. Indeed, when we explored the barriers and facilitators of just the highest quality papers, knowledge no longer featured as the main barrier or facilitator to PSB. A defining feature of the highest quality papers was the use of qualitative methods, which suggests that questionnaire methods may be overly restrictive, whereas although guided by a topic guide, the conversational and probing nature of qualitative methods allows for more spontaneous and in-depth exploration. Ultimately, these findings show the need for interventions to move beyond simple knowledge transfer, as this may fail to address the true underlying barriers to the adoption of good oral health behaviours. Indeed, a recent systematic review has explored current home-based toothbrushing interventions for parents of young children finding that there is an over-reliance on simple knowledge transfer and, although improving, a lack of theoretical underpinning and consideration of the wider context [77]. This review explains why solely focusing on knowledge transfer is unlikely to lead to effective oral health conversations. Practitioners need to listen to parents, allowing them to describe the challenges they face in order to fully understand their needs and tailor advice accordingly. This will require an approach that draws upon strong communication skills and the application of behaviour change theory to ensure we move from overloading patients with information to having a meaningful oral health conversation whereby health professionals and parents work in partnership to explore barriers to oral health care and potential solutions. Furthermore, the utility of the present review is not restricted to individual conversations, but can support other oral health interventions, such as how we train early-years professionals and the focus of public health campaigns. 

At an individual level, as with any behaviour, the individual must be motivated to perform it, and indeed the literature showed that parents who had greater motivation to brush their children’s teeth did so [37, 48, 63, 68, 78]. There are several reasons for this: one being the influence of parents highly motivated to maintain their own health [19, 21, 54, 56–60]. There was also some indication that older parents were more likely to take care of their children’s oral health [22, 56, 72]. This could be due to greater socio-economic status and/or due to gaining greater knowledge and experience over time leading to greater motivation to perform PSB. Nevertheless, the stress of daily life and busy schedules [20, 24, 33, 36, 37, 79], especially when there is more than one child in a family [60, 72, 80], can lead to conflicting priorities making undertaking PSB difficult, with evening toothbrushing being reported as particularly difficult [20, 28, 37, 62]. Parents were shown to lack skills, as well as confidence to brush their children’s teeth [35, 37, 63, 81]. Some believed that children were capable of brushing their own teeth independently [17, 18, 80]. Therefore, striking a balance between effective parental involvement in children’s toothbrushing while encouraging and teaching independence as the child matures will be an important endeavour for future PSB health promotion programmes.

It is important to acknowledge, however, that there is a wider social element to PSB at both an interpersonal (parent and child) and wider societal level (i.e. the influence of family, friends and health professionals). The lack of a child’s interest or desire for independence as well as parent’s lack of skills to encourage child’s cooperation was shown to be the main barrier at the interpersonal level [20, 37, 40–43, 45]. This was reported to be more challenging when the child was upset [26, 35]. Consequently, these findings highlight that the skills needed to effectively perform PSB were beyond simply knowing how to brush children’s teeth, and that wider parenting skills, such as behaviour management, are vital to improve performance of PSB.

At a social level, lack of support from family members was found to be the main barrier [26, 28, 35, 56]. On the other hand, receiving support from the community was reported as a key facilitator [20, 33, 37, 63]. Although, such social comparisons were found to be a hindrance as much as a help in some instances. For example, in Moore and Chestnutt [78] parents reported that they perceived the oral care they provided for their child, despite being sub-optimal (e.g. brushing once rather than twice daily) was adequate as it was better in comparison to other parents. This highlights that the wider community must be considered when delivering oral health promotion, as when used effectively the community can provide substantial influence and support to parents with young children. Nonetheless, the role of health professionals cannot be underestimated. In the current review, it emerged that parents were having difficulties accessing both dental care and information, and the conflicting health messages presented by various health professionals left parents feeling the recommendations were unrealistic and complicated [28, 30, 33]. As such, access to empathetic dentists and health educators providing advice at the early stages in a child’s life was seen as needed. Overall, the numerous barriers and facilitators identified in the current review clearly indicate that PSB is a more complex behaviour than previously perceived, and various skills and competencies beyond toothbrushing and at different socio-ecological levels (individual, interpersonal, societal and environmental) will need to be addressed by effective interventions.

Several associations were found in the literature between demographic factors, barriers and facilitators, PSB and tooth decay. Despite the wide variety of factors found to be significant within these relationships, a number of factors consistently emerged as important, including attitudes towards oral health [50, 65, 69, 71], knowledge [23, 47], perceived capability of the parent [65, 67, 70], the child’s temperament [38, 44] and family functioning [39, 41, 43]. Less commonly reported significant factors included daily routines [62], parents’ perception of level of care [57] and parents’ dental practices and care attendance [56, 71]. However, not every study found these factors to be significantly associated with tooth decay experience [55, 60]. Furthermore, demographic factors, such as ethnicity, socioeconomic status, education level, parental age, child’s age and number of children, consistently emerged as significant influences on tooth decay development, primarily through differences in attitudes and knowledge towards children’s teeth and oral health behaviours.

### Strengths and limitations

In an effort to maintain the currency of the present review, we updated the searches. Unfortunately, however, some databases that were included in the original search were unavailable when it came to updating the search; therefore, it is possible that some potentially relevant papers have not been included within the review.

Furthermore, while we identified the barriers and facilitators to PSB described in the current literature, it may be that other barriers and facilitators exist that have not previously been studied. As such, there is a need for future studies to broaden the scope of their research focus and use more open measures, for example, by using a framework (such as the TDF), upon which to structure explorations of barriers and facilitators to ensure that a full range of influences are captured as well as using inductive and deductive approaches to qualitative methods to optimise the chances of capturing new and different themes. However, a major strength of the current research is that it is the first comprehensive review of the literature regarding PSB practices. This review has synthesised the literature on barriers and facilitators related to PSB, thus providing a detailed overview of the vital determinants of PSB behaviour, and therefore the mechanisms of behaviour change to address in future interventions. Furthermore, the current review used the TDF to categorise the barriers and facilitators to PSB. This strategy was adopted to ensure consistency in the description of the construct, and thus provide a common language that can be understood within a multi-disciplinary field. No systematic review to date has used a comprehensive psychological framework to map barriers and facilitators to oral health behaviours for parents of young children. This rigour provides a methodology to support design and evaluation of future oral health interventions aimed at supporting parents, patients and carers to undertake good oral health behaviours. In addition, a quality assessment tool was used that was applicable for both quantitative and qualitative study designs. This permitted a uniform quality assessment approach that was applicable to all the included studies. The use of such a quality framework is pertinent to explore how the quality of papers influenced our findings. The rigour of the methods employed will help to improve future preventive interventions and conversations.

Due to including a wide range of study designs and specifically focussing on the barriers and facilitators to PSB, the exact nature of the relationship between PSB behaviours and tooth decay was not possible to determine in the current systematic review. In order to ascertain the effect size of PSB on tooth decay, a meta-analysis would be required with tight inclusion criteria, for example, including only experimental study designs, such as randomised controlled trials and uniform outcomes. The present systematic review provides a vital first step in this process by identifying the variety of barriers and facilitators that are associated with PSB and potentially with tooth decay.

The present systematic review is the first to assess a wide range of papers to comprehensively collate and signpost the currently existing evidence on the barriers and facilitators to PSB. Oral health conversations between dental professionals and parents that simply focus on knowledge transfer are unlikely to be effective. This systematic review provides clear evidence of a wide range of barriers and facilitators of PSB for young children. Moreover, these barriers and facilitators can act at personal, interpersonal, family, community and societal levels. Understanding these oral behaviours requires a tailored approach that is cognisant of the many daily challenges families face and draws upon strong communication skills and the application of behaviour change theory. As such, training in these areas is highly recommended in conjunction with interventions which are robustly developed and evaluated following complex intervention methodology. With finite funding, the review helps to prioritise the focus of supporting resources based on their frequency reported in the literature.

## Supplementary information

ESM 1 (DOCX 15 kb)

ESM 2 (DOCX 72 kb)

ESM 3 (DOCX 81 kb)

ESM 4 (DOC 64 kb)

## Footnotes

1 Not all the included studies assessed or reported associations between barriers/facilitators, PSB behaviour and/or tooth decay.

## Declarations

### Disclaimer

The views expressed in this publication are those of the author(s) and not necessarily those of the NIHR, the NHS or the Department of Health and Social Care.
