# Supplementary material for: Facilitators and barriers to home-based toothbrushing practices by parents of young children to reduce tooth decay: a systematic review
Source: Clin Oral Investig. 2021 Mar 20;25(6):3383–93. doi: 10.1007/s00784-021-03890-z (PMC8137613; doi:10.1007/s00784-021-03890-z)
Supplement: Supplementary file 1 — (DOCX 15 kb) [file 784_2021_3890_MOESM1_ESM.docx]

Supplementary materials: Example search strategy

MEDLINE (OVID)

1. Toothbrushing/ed, is, mt, nu, px, sn, td, ut [Education, Instrumentation, Methods, Nursing, Psychology, Statistics & Numerical Data, Trends, Utilization]

2. toothbrush*.mp. [mp=title, abstract, original title, name of substance word, subject heading word, keyword heading word, protocol supplementary concept word, rare disease supplementary concept word, unique identifier]

3. (brush* adj4 teeth).mp. [mp=title, abstract, original title, name of substance word, subject heading word, keyword heading word, protocol supplementary concept word, rare disease supplementary concept word, unique identifier]

4. (brush* adj4 tooth).mp. [mp=title, abstract, original title, name of substance word, subject heading word, keyword heading word, protocol supplementary concept word, rare disease supplementary concept word, unique identifier]

5. 1 or 2 or 3 or 4

6. *Oral Health/ed, mt, sn, td [Education, Methods, Statistics & Numerical Data, Trends]

7. *Oral Hygiene/ed, mt, nu, px, sn, td, ut [Education, Methods, Nursing, Psychology, Statistics & Numerical Data, Trends, Utilization]

8. 5 or 6 or 7

9. *Dental Care for Children/is, ma, mt, nu, og, px, st, sn, td, ut [Instrumentation, Manpower, Methods, Nursing, Organization & Administration, Psychology, Standards, Statistics & Numerical Data, Trends, Utilization]

10. parent*.mp. [mp=title, abstract, original title, name of substance word, subject heading word, keyword heading word, protocol supplementary concept word, rare disease supplementary concept word, unique identifier]

11. carer*.mp. [mp=title, abstract, original title, name of substance word, subject heading word, keyword heading word, protocol supplementary concept word, rare disease supplementary concept word, unique identifier]

12. *Parents/ed, px [Education, Psychology]

13. *maternal behavior/ or *parent-child relations/ or *parenting/ or *paternal behavior/

14. 9 or 10 or 11 or 12 or 13

15. 8 and 14
