# Supplementary material for: Facilitators and barriers to home-based toothbrushing practices by parents of young children to reduce tooth decay: a systematic review
Source: Clin Oral Investig. 2021 Mar 20;25(6):3383–93. doi: 10.1007/s00784-021-03890-z (PMC8137613; doi:10.1007/s00784-021-03890-z)
Supplement: Supplementary file 2 — (DOCX 72 kb) [file 784_2021_3890_MOESM2_ESM.docx]

*Table 1: Summary of studies reporting on barriers (B) and facilitators (F) to parental supervised toothbrushing (PSB)*

| **Paper** | **Quality** | **Country** | **Design** | **Number of parents** | **Age & gender group** | **Ethnicity** | **Outcome measures** | **Barriers (TDF construct)** | **Facilitators (TDF construct)** | **Influential demographic factors** | **Association between B/F with any changes (outcome measure)** |
| --- | --- | --- | --- | --- | --- | --- | --- | --- | --- | --- | --- |
| Adair et al. (2004) | 19 | UK | Quantitative (international study) | 2, 822 parents | Under 20 to over 50  84% of respondents were the child’s mother, 13% father, 2% grandparent and the remaining 1%, other relatives or guardians  Children aged 3-4 years | 17 countries (Europe, Africa, America, Asia) | Attitudes towards oral health | Ethnic attitudes towards importance of TB  (Beliefs about consequences)  Lack of control over child’s TB in low socioeconomic status families (Beliefs about capabilities) | Parental self-efficacy in establishing TB behaviour (Beliefs about capabilities)  Positive parental attitudes towards TB (Beliefs about consequences) | Higher education level of mother | Significant difference between caries and non-caries groups and education level of mother with attitudes towards importance and intention to brush child’s teeth and their efficacy to TB (p<0.001) |
| Adiatman et al. (2017) | 13 | Indonesia | Quantitative (Questionnaire) | 295 | All female  Mean+ _SD=31.09+_5.80  Children < 6  150 boys and 145 girls | NR | Mother’s habit of helping their children brush their teeth twice a day  & early childhood caries (dmft) | Negative attitudes towards the habit of helping children brush their teeth twice a day  (Beliefs about consequences)  Negative opinion of people involved in the  habit of helping children brushing their teeth twice a day  (Social influences)  Lack of ability to help children brush their teeth twice a day  (Beliefs about capabilities)  Lack of intention to help children brush their teeth twice a day (Motivation and goals)  Lack of habit of helping children brush their teeth twice a day (Nature of behaviour) | Positive attitudes towards the habit of helping children brush their teeth twice a day  (Beliefs about consequences)  Positive opinion of people involved in the habit of helping children brushing their teeth twice a day (Social influences)  Ability to help children brush their teeth twice a day (Beliefs about capabilities)  Intention to help children brush their teeth twice a day (Motivation and goals)  Habit of helping children brush their teeth twice a day (Nature of behaviour) | NR | An increase in attitudes and social norms increases intentions, in turn increasing the habitual behaviour of helping children brush their teeth twice a day (p<0.001).  No significant correlation between the habitual behaviour of helping children brush their teeth twice a day and early childhood caries and plaque (p>0.05). |
| Akpabio et al. (2008) | 23 | USA (Michigan) | Quantitative (structured interview) | 105 | All female  19-54 years (mean: 29.96)  Younger children | Not reported (NR) | Knowledge toward promoting good oral health for children | Lack of knowledge on TB (Knowledge) | Parents health behaviour (frequency of TB and flossing) (Social influences)  Parents dental anxiety (Emotion)  Knowledge concerning the consequences of poor oral health (Knowledge/Beliefs about consequences) | Parents’  income  Higher number of children | Increased income was associated with greater knowledge about oral health care utilization p=0.003  There was a trend between knowledge regarding the prevention of oral disease and the number of children in a family (p=.055) |
| Amin et al. (2009) | 19 | Canada (English and Chinese speaking) | Qualitative (interview) | 19 | 14 mothers and 5 fathers- 26 to 45 years  10 Children 2.5 – 6 years (mean 3.9) | NR | Parent’s oral health knowledge and barriers and facilitators to oral health behaviour | Difficulties accessing dental service (availability and cost) (Environmental context and resources)  Recommendations unrealistic and complicated (Beliefs about capabilities)  Health-related conflicting information (Social influences)  Stress of life (unemployment, finance and family issues) (Environmental context and resources)  Child’s behaviour (Behaviour regulation)  Lack of time and parent availability (Environmental context and resources)  Other caregivers/siblings involvement/influence  (Social influences) | Practical advice and demonstrations (Skills)  Community support  (Social influences) | NR | NR |
| Ashkanani et al. (2013) | 27 |  | Quantitative (Questionnaire) | 334 parents | Parents 70% between 20-40 years old  Children 0-5 years old | NR | Parents knowledge, attitudes and practices towards  the oral health of their preschool children | Lack of knowledge about when to start brushing child’s teeth (Knowledge)  Having more than 5 children (Environmental context and resources) | Good knowledge of when to start brushing child’s teeth (Knowledge) | High education level  Being an older parent (over 40) | Mothers of children aged 0–1 years old had  better knowledge of the ideal time  to start brushing child’s teeth compared to mothers of older children (p = 0.000)  Parents education level had a  significant effect on their practices (p = 0.017)  Parents with more than 5 children were less likely to brush their children’s teeth 2-3 times a day (p = 0.030)  Older parents (over 40 years) were more committed to brushing child’s teeth (p = 0.040) |
| Ayoub (2017) | 27 | Greater Boston, USA | Quantitative (questionnaire) | 941 mother-child dyads | Mothers  Children 1-5 years | Hispanic/Non-hispanic | Children’s brushing frequency | NR | Maternal knowledge about children’s oral health (Knowledge)  Maternal self-efficacy (Beliefs about capabilities)  Maternal self-motivation (Motivation and goals)  Dental visit within past year (Knowledge/Beliefs about consequences) | Ethnicity  Mother’s age  Child’s age | 65.3% of the children had their teeth brushed twice or more a day and 71.6% of children’s brushing was supervised.  Maternal self-efficacy and self-motivation  significantly predicted children’s brushing frequency (OR=10.51; 95% CI= 6.98-15.81 and OR=7.41; 95% CI=2.63-20.85 respectively).  Maternal knowledge about child’s oral health was not significantly  associated children’s teeth being brushed twice or more a day (OR= 1.33, 95%  CI= 0.96-1.84). |
| Baginska et al. (2012) | 10 | Poland  (Bialyslok) | Quantitative (questionnaire) | 140 | Mothers  Children 3-4 years | NR | NR | Lack of knowledge of importance of primary dentition (Knowledge)  Not realizing difficulty children have TB  (Beliefs about capabilities) | Oral health education and promotion  involving parents, dentists, general practitioners and paediatricians (Knowledge/Social influences) | NR | More than 20% did not know they should assist children with TB until 10 years of age.  20% felt there was no need to assist with TB.  A number of parents did not realise that plaque removal is difficult for a child. |
| Begzati et al. (2014) | 15 | Kosovo | Quantitative (structured interviews) | 664 | Children preschool age  326 females  Parents 29.33+/_4.92  Gender NR | NR | Knowledge and habits regarding oral health practices | Lack of knowledge regarding oral health protection (Knowledge)  Lack of effort regarding preventive oral health behaviours (assisted TB, proper TB technique and use of FTP) (Motivation and goals) | Health education (pre- and post-natal) (Knowledge) | NR | Children of parents with a higher education level (high school or university) had significantly lower dmft (p<0.001) |
| Bennadi et al. (2014) | 13 | India | Quantitative (cross-sectional questionnaire) | 248 | All mothers  Age NR  Children3-6 years old | NR | Knowledge of TB practices and TP utilisation | Lack of knowledge regarding commencement of TB and fluoride content, fluorosis, rinsing after TB and confusion over which TP to select (Knowledge) | Knowledge of risks associate with fluoride (Knowledge)  Being aware of the preventive role of TB and fluoride  (Knowledge) | NR | NR |
| Blinkhorn et al. (1978) | 8 | UK | Quantitative  (Structured interview) | 288 parents | All mothers  Age NR  Children 2-4 years old | NR | Child's TB behaviour and mother's expectations concerning the social norms related to TB  for children | Lack of social support or family support (Social influences)  Health professional failed to demonstrate to mothers how to assist with TB  (Skills)  Lack of interest concerning oral health (Motivation and goals) | Parents regular dental attendance patterns (Social influences)  Presence of dental health educators (Social influences) | Deprivation  High social class of parents  Older age of parents | Older mothers taught routines to family members more than younger mothers (p<0.01)  More mothers who were regular attenders taught their children to brush than those who were irregular  attenders (p<0.01)  Children who did not own a toothbrush were from mothers more likely to live in working class areas (p<0.01) |
| Blinkhorn et al. (2001) | 10 | UK | Quantitative (questionnaire) | 268 mothers and their children | NR  1-6 years old  Median:4 | NR | Knowledge and attitudes regarding oral health and observed parental TB | Children want to be independent and copy parents (Behaviour regulation/Social influences)  Lack of practice of TB knowledge (recommendations)  (Skills)  Lack of knowledge about F (Knowledge) | Make TB daily routine (Nature of behaviour)  Empathetic health professional (Social influences)  Demonstration and practical assistance (Skills) | Low socioeconomic status (SES) | A greater proportion of parents who claimed to use the correct amount of TP and stand behind child when TB were observed to actually perform these behaviours *(p<0.001)* |
| Boustedt et al. (2019) | 13 | Sweden | Quantitative (questionnaires) | 336 | Parents age and sex NR  Children: 52% boys and 48% girls | 93% Swedish | Toothbrushing behaviour & caries | Child seeks to avoid toothbrushing; toothbrushing performed with difficulty  (Skills/Behaviour regulation)  Parent unable to brush, the  child averts toothbrushing  (Skills/ Behaviour regulation) | Cooperative child  (Behaviour regulation) | NR | Minor (i.e., toothbrushing performed with difficulty with avoidant child) and major (i.e., parent unable to brush, so child averts toothbrushing) problems with brushing at age of 2 and 3 years old positively associated with caries at 5 years old (p<0.01) |
| Bozorgmehr et al. (2013) | 23 | Iran | Quantitative (questionnaire) | 222  children | 89% mothers  9% fathers  1.3 care giver or grandparents  23-45  Mean age:33  Children 5 years old | NR | Health behaviour and TB habits of parents and children | Poor oral health behaviours of parents  (Social influences)  Cost of dental provisions (Environmental context and resources) | NR | NR | NR |
| Broder et al. (2006) | 19 | America | Quantitative (cross-sectional - structured interview) | 60 | All fathers  Age (Mean 30.8 (Sd 8.6)  Children’s mean age 2.1 years (Sd 0.9) | African-American | Cognitive and behavioural factors influencing oral health behaviour | Lack of parental self-efficacy to stop behaviours that put children at risk of cavities (Beliefs about capabilities) | NR | NR | NR |
| Carvalho et al. (2014) | 28 | Brazil | Quantitative (structured interview) | 2,145 parents | Parents 15-40+ years  Children1-5 years old | NR | Caries | NR | NR | Low education level | Not assisting the child in toothbrushing significantly related to caries status (d_1_efs, p < 0.0001)  Caries outcome was significantly  associated with not assisting the child in toothbrushing  for children of mothers with 4 years of education  (< 4 years education vs. > 4 years education, OR = 2.35; 95% CI 1.69–3.25; p < 0.0001) |
| Chen et al. (2014) | 28 | Taiwan | Quantitative (cross-sectional questionnaires) | Immigrants= 148  Native = 426 | The  average ages of the immigrant mothers: 29.3  native mothers: 36.2 years  Children 4-6 years old | Vietnamese and Indonesian as immigrants  Native: Tiwanian | Caries related knowledge and attitudes  Maternal oral health behaviours | NR | Higher level of caries-related knowledge (Knowledge) | Ethnicity  Immigration/racial differences (education and income) | There was a significant difference between immigrant and native mothers in terms of  knowledge, attitudes toward oral hygiene,  and parental indulgence attitudes  (p < .001).  Nearly 50% of immigrant mothers  are uncertain about using FTP  62.73% of native mothers chose FTP  There were significant differences between immigrant and native mothers in terms of maternal behaviors  toward cleaning teeth of children (e.g. age of commencement of PSB)  62.33% immigrant mothers began TB at 1year of age or older    61.73% native mothers  began TB under 1 year of age |
| Chhabra et al. (2012) | 15 | India (Ghaziabad) | Quantitative (comprehensive questionnaire) | 620 parents | 24-30 years 620 (504 female parents and 116 male parents)  Children 1-4 years | NR | Parental knowledge and awareness regarding oral and body health | Lack of knowledge and awareness of importance of the primary teeth and TB recommendations (Knowledge)  Belief children capable of brushing own teeth by age of 3 years (Beliefs about capabilities)  Fear of dental treatment (Emotion) | NR | NR | NR |
| Collett et al. (2016) | 14 | USA | Quantitative (observational study) | 93 mothers | Mother’s age NR  Children: under 24months (n=7);  24-42 months (n=43);  Above 42 months (n=43)  40 girls and 53 boys | Non-white or Hispanic (n=27) and White, non-Hispanic (n=29) | Toothbrushing Observation System (TBOS)  &  dmfs | Punitive parenting behaviours  (Skills)  Child non-compliance/refusal  (Behaviour regulation) | Adaptive parent behaviour management strategies  (Skills)  Child compliance  (Behaviour regulation) | NR | Higher parent TBOS scores (indicative of more adaptive parenting) and longer duration of parent-led toothbrushing was inversely associated with dmfs (IRR = 0.53, 95% CI = 0.24, 0.81 and IRR = 0.88, 95% CI = 0.77, 1.00 respectively) |
| Daly et al. 2016 | 17 | USA | Questionnaire | 1323 parent/infant pairs | 94% mothers, 6% fathers, <1%  grandmothers as legal guardians, and <1% other legal  guardians. Age, years, mean ± SD 28.7 ± 6.0  Children n = 1323  Gender  Male 674 (50.9)  Female 649 (49.1)  Age, months, mean ± SD 11.4 ± 2.0 | Parents  Black/African American 527 (39.8)  Native American 7 (0.5)  Asian 30 (2.3)  White 646 (48.8)  More than one race 65 (4.9)  Other 48 (3.6)  Ethnicity  Hispanic 145 (11.0)  Children Black/African American 510 (38.6)  Native American 4 (0.3)  Asian 21 (1.6)  White 581 (43.9)  More than one race 165 (12.5)  Other 42 (3.2)  Ethnicity  Hispanic 177 (13.4) | Parental care of child’s oral health compared to medical health and factors associated with parental perceptions of how well they do in taking  care of child’s oral health | Perceive  their job of taking care of the teeth and/or gums as poor  (Beliefs about capabilities) | Perceive they provide good care for child’s teeth  (Beliefs about capabilities)    Parents who take good care of own oral health  (Social influences) | NR | Parents  who  perceive they provide good care for their children’s oral health care brush their children’s teeth daily (p<.001) |
| Danila et al. (1995) | 21 | Romania | Quantitative (structured interview) | 322 mothers and children | Mothers  Children 7 years old | NR | Parental knowledge, attitudes and behaviour; and sources of oral health information, evaluation of child’s dental health, number of children and education level | NR | Attitude that children need help with brushing (Beliefs about consequences) | NR | NR |
| Davidovich et al. (2013) | 12 | Israel | Quantitative  (cross-sectional structured questionnaire) | 719 | NR  Children18-82 months | Ethiopian and Israeli | Presence of TB and TP at home, the help children received during TB  Frequency of child’s TB | NR | Education targeting immigrants (Knowledge) | Immigration and cultural background | More Israeli children owned TB (p=0.0001) and used TP (p=0.004) than Ethiopian children  Israeli parents helped with TB more than Ethiopian parents |
| Duijster et al. (2014) | 34 | The Netherlands | Quantitative (Questionnaires) | 630 children | Children 5-6 years old | Dutch, Moroccan, Turkish and Surinamese | Sociodemographic characteristics, oral hygiene behaviours and family  functioning | Poorer functioning families (clinical and subclinical) (Social influences/behaviour regulation) | Normal functioning families (Social influences/behaviour regulation) | NR | Supervised brushing and rebrushing significantly associated with dmft status (β=0.94, CI 0.53-1.34, p<0.001; and β=0.41, CI 0.16-0.66, p<0.01)  There were significant differences across families of differing functioning levels and TB behaviour (commencement, frequency and supervision/rebrushing, p<0.05) |
| Duijster (2015) | 29 | Netherlands | Qualitative (focus groups) | 39 parents | Children 7.2 ± 0.5 years  31 Female 8 Male | 2x focus groups High SES Dutch  2x focus groups low SES Dutch  1x Moroccan  1x Turkish | Parental views on influences on children’s oral  health behaviours  parents’ views on limitations  and opportunities for professional oral health support | Belief that oral hygiene efforts could not fully prevent their child from getting tooth decay (external locus of control) (Beliefs about capabilities)  Time constraint/busy schedule (Environmental context and resources)  Lack of knowledge (Knowledge) | Parents perception of twice daily tooth brushing as a generally  accepted standard of behaviour (social norm) (Social influences)  Perceived importance of TB  (Beliefs about consequences)  Parental confidence in TB  (Beliefs about capabilities)  Parenting strategies maximizing compliance using positive reinforcement  or by turning tooth brushing  into an easy/enjoyable activity (Behaviour regulation)  Tooth brushing routines and habituation (Nature of behaviour)  Role modelling - brushing their own  teeth in their child’s presence (Social influences) | NR | NR |
| Duijster et al. (2015) | 31 | Netherlands | Quantitative (Cross-sectional –questionnaire and structured video observations) | 92 parent-child dyads | Parents:  74% biological mother, 26% biological father  Children: 5-6 years old, 38 males, 54 females | Dutch, Moroccan, Turkish | Sociodemographic characteristics, oral health behaviors, parents’ dental self-efﬁcacy, locus of control (LoC), parenting practices and family functioning. | “Ineffective parenting”, i.e., lower levels of positive  involvement, encouragement and problem-solving (Skills/Behaviour regulation/Memory, attention and decision processes/Emotion) | Internal locus of control (Beliefs about capabilities)  Higher dental self-efficacy (Beliefs about capabilities)  Positive parenting practices, i.e., positive involvement, encouragement and problem-solving (Skills/Behaviour regulation/Memory, attention and decision processes/Emotion) | Lower social class | Positive parenting practices of encouragement and problem-solving significantly associated with caries-free children (p=<0.001) |
| Ekman et al. (1981) | 18 | Sweden | Quantitative (questionnaire) | 116 children and their parents | Parents 22-50 years  Gender NR  Children 4-6 years old (mean age: 5)  47 boys | Finnish and Swedish | Oral health attitudes of parents | NR | Parent good dental health (Social influences)  Positive attitude to dental health (Beliefs about consequences)  Early provision of information and motivation (Knowledge/Motivation and goals) | Language barriers | NR |
| Finlayson et al. (2005) | 22 | Detroit, USA | Quantitative (questionnaires) | 719 | Parents: NR  Children 1-5 years old  Gender NR | African- Americans | Frequency of TB | NR | High confidence in ensuring teeth brushed (Beliefs about capabilities) | NR | Children’s of mothers with higher confidence in their ability to make sure the child’s teeth are brushed at bed time were more likely to brush their teeth more frequently (r=0.18, p<0.0001) |
| Hamilton et al. (2018) | 28 | Australia | Quantitative (longitudinal, questionnaire) | 281 parents | Children 2-5 years old  Mean parental age 37.05 | Caucasian/ non-caucasian | Effect of intention via self-efficacy and planning, and action control on PSB | NR | Perceived ability to control child’s toothbrushing habits – high self-efficacy (Beliefs about capabilities)  Intention to undertake PSB through planning and action control (Motivation and goals) | Lower parental age | Significant indirect effects from intention via self-efficacy and action  control (p<.01), and intention via planning and action control (p=.05) on parental supervised toothbrushing |
| Huebner et al. (2010) | 39 | USA | Mixed (interview with some quantitative data provided) | 44 parents | Children 1-5 years old | Most Caucasian | Parents' motivation, support, and barriers to  twice daily TB | Negative oral health beliefs (Beliefs about consequences)  Lack of social norms and support  (Social influences)  Emotional reaction to upset child (Emotion)  Poor self-standards for TB  (Social influences)  Lack of self-efficacy to reduce barriers to TB  (Beliefs about capabilities)  Lack of skills to make TB routine  (Skills/Nature of behaviour)  External supports (uncooperative child and lack of time) (Behaviour regulation/ Enviromental context and resources) | Positive oral health beliefs regarding importance of TB  (Beliefs about consequences)  Benefits of TB and parental duty (Social/professional role and identity)  Social norms, primarily social support (Social influences)  Emotional reactions to consequences of not TB (Emotion)  High self-standards for TB  (Social influences)  Self-efficacy regarding routine setting (Beliefs about capabilities/ Nature of behaviour)  Skills to encourage cooperation (Skills)  External supports (cooperative or independent child) (Behaviour regulation) | NR | NR |
| Janson et al. (1993) | 10 | Amman | Quantitative (structured interview) | 255 | Children 6 months to 6 years  151 boys  Parents: all mothers | NR | Tooth cleaning habits | NR | NR | High level of education | Higher educated mothers (diploma or university degree) cleaned their children’s teeth more often (25%) than others (12%) p< 0.01. Yet, the prevalence of caries in their children was the same (77%) as children of the mothers with less than 4 years schooling (80%) |
| Lencova et al. (2013) | 25 | Czech | Quantitative  (Questionnaires) | 796 parents | Children 3-5 years old  Gender NR  Parents NR | NR | Parental oral health beliefs | Lack of self-efficacy to TB (Beliefs about capabilities)  Poor attitudes towards TB and outcomes (Beliefs about consequences) | Parents’ willingness/determination to encourage and supervise children during TB (Motivation and goals)    Parental self-efficacy to undertake TB (Beliefs about capabilities)  Positive attitudes of people around towards TB (Social influences)  Awareness that it is parents responsibility to control caries risk in their child (Social/professional role and identity) | SES  Level of education | There was a significant difference in terms of TB – perceived significance and parental efficacy, external caries control and internal caries control  between different self-perceived SES and the  education level of the mothers  (p≤0.005).  Fathers´ education did not significantly influence toothbrushing behaviour  (p=0.11), but did influence external  (p=0.00) and  internal caries control (p=0.01). |
| Marshman et al. (2016) | 32 | UK | Qualitative  (semi-structured interviews) | 27 parents | Children < 7 y Age NR  Parents Age NR  22 mothers 2 fathers 3 grandmothers | White British, Pakistani, Polish, Russian | Parents experience of tooth brushing with children | Child’s behaviour – resistance to toothbrushing (Behaviour regulation)  Child’s behaviour – tiredness (Environmental context and resources)  Forgetting to brush (Memory, attention, and decision processes)  Perceived lack of control over child’s behaviour (Beliefs about capabilities)  Lack of parenting skills in managing behaviour (Skills/Behaviour regulation)  Parent assumes monitoring role (Social/professional role and identity)  Demands on attention – e.g., multiple children, morning/bedtime routines, breaks in routines, ‘stressful’ circumstances (Environmental context and resources/Nature of behaviour)  Lack of capacity to prioritise supervision of toothbrushing (Motivation and goals)  Lack of parenting skills in maintaining routines (Skills/Nature of behaviour) | Social support from TV, grandparents, friends and health professionals (Social influences)  Knowledge of appropriate health behaviours and level of involvement needed in child’s toothbrushing (Knowledge)  Attitudes regarding consequences of tooth decay and personal negative dental experience (Beliefs about consequences/Emotion)  Intention to brush child’s teeth (Motivation and goals)  Skills to control and manage behaviour (Skills/Behaviour regulation)  Skills to develop and maintain a routine (Skills/Nature of behaviour) | NR | NR |
| Mofidi et al, (2009) | 21 | USA | Qualitative (focus groups) | 22 parents, and 13 pregnant women | Parents mean age 37, pregnant women’s mean age 38.1  All parents females except one: male  Pre-school aged children | White, African American, Latino and American Indian | Opinions, values, current practices and recommendations on the oral health of young children and pregnant women | Lack of understanding of the importance of primary teeth and oral health care of their child  (Beliefs about consequences)  Busy schedules (Enviromental context and resources)  Conflicting life demands (Enviromental context and resources)  Uncooperative child (Behaviour regulation)  Lack of knowledge about how to brush a young child’s teeth (Knowledge) | Improving knowledge, skills and attitudes (Knowledge/Skills/Beliefs about consequences)  Cultural sensitivity when promoting oral health care (Social influences) | NR | NR |
| Nagarajappa et al. (2013) | 23 | India | Quantitative (cross-sectional structured questionnaires) | 470 | Children 6 months to 3 years old  G: NR  Parents: 20- <35  63.6% females | NR | Attitudes and practices towards TB | NR | Greater knowledge, attitudes and practices associate with parental age and being the mother (Knowledge/Beliefs about consequences/Skills) | SES  Parental age | Parents aged 25-29, mothers and parents of a higher SES had significantly higher knowledge, attitude and practice scores than other groups |
| Naidu et al. (2008) | 15 | West indies (Trinidad and Tobago) | Quantitative (questionnaire) | 138 parents or guardians | Parents age 18-64  102 (73.9%) female  Pre-school children | African, Indian, Mixed, Others/not given | Parental knowledge and attitudes towards oral health of young children | Lack of knowledge regarding position to adopt while TB, amount of TP and fluoride to use, how to  if TP contains fluoride  (Knowledge) | NR | NR | 67% with primary education would brush from in front compared to 17% with secondary education  More parents with a secondary education did not know the best position of TB compared to those with a primary education (p<0.05) |
| Naidu et al. (2012) | 24 | Spain | Qualitative (interview) | 18 parents and caregivers | Female (89%) with a mean age of 28 (ranging from 23 to 49 years-old) | 67% Indian 28% African or 5% Mixed ethnicity  Pre-schoolers | Oral health beliefs attitudes and behaviours of caregivers | Difficulty in achieving night-time brushing (Environmental context and resources)  Confusion over fluoride (Knowledge)  Encouraging child to brush themselves to build up confidence (Behaviour regulation)  Reliance on friends and family for dental health information (Social influences)  Difficultly accessing oral health information (Environmental context and resources)  Lack of support from partner  (Social influences) | NR | NR | NR |
| Narksawat et al. (2011) | 21 | Thailand | Quantitative (Structured interview) | 664 parents and children | NR  349 boys (52.5%)  Children’s mean age: 3.7+-0.8 | NR | Important oral hygiene practices of parents | Lack of access to dental services (Environmental context and resources)  Lack of parental knowledge (Knowledge) | Cleaning child’s teeth when bathing them  (Nature of behaviour) | NR | There was a greater likelihood of children developing  caries in those parents who did not always clean their children’s teeth every time when bathing them (OR = 2.1;  95% CI = 1.4-3.1) |
| Paunio et al. (1994) | 14 | Finland | Quantitative  (structured questionnaire) | NR | All mothers- age NR  Children 3 years old | NR | TB frequency, and the explanatory risk factors | Lack of knowledge about importance of primary teeth (Knowledge)  Younger mothers less engaged in self-care and less willing to share time and energy with children) (Social influences/Motivation and goals) | Mother’s healthy manner of life (Social influences) | Mother’s age | NR |
| Petersen et al. (1992) | 16 | Quantitative (Structured and semi-structured questionnaires) | Denmark | 220 children | 88% female  Age NR  Children 6 years old | NR | Knowledge, attitude, oral health behaviour | NR | Knowledge about the causes and prevention of dental diseases (Knowledge)  Oral health information provided by dentists and magazines (Social influences)  Positive dental attitudes (Beliefs about consequences)  Greater support for first born child (Social influence) | Income  High education level of parents | Family income (p<0.05), and education  of parents (p<0.01) were significant predictors of dental caries |
| Petersen et al. (1998) | 17 | China | Quantitative (structured interview) | 691 parents  382 children | NR  Children6 years old | NR | Oral health  knowledge, and attitudes towards prevention of oral  disease | Lack of knowledge and poor attitudes about prevention and supervision (Knowledge/Beliefs about consequences) | Educational programme (Knowledge) | NR | NR |
| Pine et al. (2004) | 17 | UK | Quantitative (Cross-sectional - questionnaire) | 2,822 children/families | Children aged 3 or 4 years | Europe, north America, Africa and Asia | Parental beliefs, attitudes and behaviours towards child’s oral health. | Cultural/ethnic attitudes (Beliefs about consequences) | Positive parental attitudes regarding perceived ability to brush child’s teeth (Beliefs about consequences/Beliefs about capabilities) | NR | Brushing before age 1, brushing twice a day and adult involvement in brushing doubled the odds of being caries-free. The most important predictor of caries was parents’ perceived ability to incorporate regular TB into the child’s daily routine. This factor was particularly prominent in disadvantaged communities. |
| Pisarnturakit et al. (2012) | 22 | Thailand | Quantitative (questionnaire) | 254 parents | 78.4% were female. The mean age 32.4 years with a SD of 8.7 years.  Kindergarten children | NR | Beliefs regarding preventive practices of dental caries among caregivers of young children. | Cost of dental care materials (Environmental context and resources)    Difficulty brushing child’s teeth (Behaviour regulation)  Concern over causing pain while TB (Emotion)  Lack of knowledge of recommendations (frequency of TB) (Knowledge)  Lack of support from family members (Social influences) | Positive attitudes regarding consequences of TB (e.g. better sleep) (Beliefs about consequences) | NR | NR |
| Prowse et al. (2014) | 19 | Winnipeg, Manitoba, Canada | Qualitative (focus groups) | 40 parents and caregivers | Childrenunder 6 years old | Aboriginal, Hutterite, immigrant (Africa and Western Asia including  Congo, Eritrea, Nigeria, and Sudan), and refugee (Africa, the Middle East, and Western Asia  including Chad, Congo, Ethiopia, Iraq, Morocco, Nigeria,  and Somalia) | Views on oral health in early childhood and early childhood caries. | Uncooperative child (Behaviour regulation)  Lack of time (Environmental context and resources)  Cost and availability of dental provision (Environmental context and resources) | Learning from family members and friends  (Social influences)  Learning from community-based health programmes (Knowledge)  Importance placed on oral hygiene by faith  (Social influences) | NR | NR |
| Rahbari and Gold (2015) | 12 | USA  (Florida) | Quantitative (questionnaire) | 103 participants  48 first-time pregnant women  55 mothers (8 of whom were also pregnant) | All female  Age range: Under 18 to 36 years and older (65% aged between 21-30)  Children under 6 years old | NR | Oral health knowledge and behaviours | NR | Mother’s oral hygiene behaviours (Social influences) | NR | Significant positive relationship between the TB frequency of the toddler performed by the mother and mother’s TB frequency (r^2^=0.29; p=0.04) and mother’s self-reported oral health rating (r^2^=0.29; p=0.03) |
| Reisine et al. (2009) | 19 | USA | Quantitative (questionnaires) | 52 | All males  18-70 years  Children < 6 years | American African | Self-efficacy in TB | Lack of knowledge of importance of brushing primary teeth using fluoride (Knowledge) | High self-efficacy to TB (Beliefs about capabilities) | NR | NR |
| Senesombath et al. (2010) | 17 | Thailand | Quantitative (structured interview) | 400 | 283 females  Age NR  Children36-47 months  196 boys | NR | Oral hygiene practices for their children | Lack of oral health knowledge (Knowledge) | Oral health education to parents in early childhood (Knowledge) | NR | NR |
| Spitz et al. (2006) | 17 | Iowa, USA | Quantitative (Dental records) | 629 children | mothers  Children0-4 years old | NR | Perception of child’s temperament and risk factors for early childhood caries | Having a child with a difficult temperament (Behaviour regulation) | Having a child with an easy temperament (Behaviour regulation) | NR | Children categorised as having a ‘difficult’ temperament were more likely to have their teeth brushed daily (p = .002), whereas children categorised as having an ‘easy’ temperament were more likely to have their teeth brushed twice a day (morning and night, p=.006)  No significant difference between ‘easy’ and ‘difficult’ children in terms of cavitated lesions (p=.269) |
| Sujlana and Pannu, (2015) | 14 | India | Quantitative (Cross sectional) | Parents of 400 children | 5 years | NR | Prevalence of dental caries.  Influence of socio-demographic and family-realted factors on caries status and oral health behaviours | Lax attitude towards child’s toothbrushing (Beliefs about consequences)  Less than twice daily brushing of parents’ teeth (Social influences) | Parents’ brushing own teeth twice daily (Social influences) | Mother’s level of education  Number of siblings | Significant association between a higher level of mothers' basic education and dmft = 0 (p < 0.001)  A higher number of siblings significantly associated with higher caries  status (p = 0.04)  Higher brushing frequency  significantly associated with dmft = 0 (p< 0.004).  Significantly  reduced dmft score in children where parents supervised brushing (p < 0.001).  No significant association between parental attitude towards dental decay and dmft > 0  Parents who brushed teeth twice daily had higher chances of having children with dmft = 0 (p < 0.001).  No significant  differences between children with dmft > 0 and those with dmft = 0 regarding parents' dental care seeking  behaviour. |
| Sun et al. (2017) | 29 | China | Quantitative (Cross-sectional) | 2795 fathers;  6734 mothers; and 193 other relatives  9722 children | Parent  2195 <30 years old;  6937 30-39 years old;  590 40+ years old  Children 5 years old | Han  or minority ethnic group | Association between early life factors and dental caries | Toothbrushing not a  social norm (Social influences) | NR | Birth order (toothbrushing commenced aged 1 year or below for firstborn)  Small families  Older parents  Parent education  Parental wealth  Urban location | Significantly higher dmft in children commencing toothbrushing after 1 year of age than those who brushed aged 1 or below, with dmft increasing each year toothbrushing delayed  (p < 0.001) |
| Sutthavong et al. (2010) | 14 | Thailand | Quantitative (cross-sectional structured interview) | 138 | NR  Children2-12 years old | NR | Knowledge, attitude and practices towards their  children | NR | Early prevention program with multiple health professional involved (Knowledge/Social influences)  Promotion of self-responsibility and awareness (Knowledge/Social/professional role and identity) | NR | No association was observed between parental knowledge and attitude towards dental care with dmft p < 0.824 and DMFT p < 0.686 and parental practices with dmft p < 0.665 and DMFT p < 0.560. |
| Szatko et al. (2004) | 23 | Poland | Quantitative (cross-sectional questionnaire) | 1033 mothers | NR  Children3 years old | NR | Oral health –related knowledge, habits and educational levels of their mothers | Lack of knowledge on TB and F (Knowledge) | Good oral health related knowledge (Knowledge)  Including information in magazine (Knowledge) | Higher level of education | There was an association between higher education level of mothers and supervised TB  Higher education level of mother was associated with a lower level of caries in children (p<0.001)  As mothers oral health knowledge increased, mean dmft of children decreased (p<0.001) |
| Tiberia et al. (2007) | 18 | Canada | Quantitative (survey) | 139 parents  128 children | Parents NR  Children’s mean age: 3.05  Gender NR | (77%) were Caucasian; (b) 7% (N=9) were First Nation; (c) 2% (N=3) were Asian; (d) Filipinos comprised 4% (N=6); and (e) 2% (N=2) were East Indians | Risk factors for ECC | Uncooperative child (Behaviour regulation) | NR | NR | Having problems brushing (*P*=.05),(ß=1.13) contributed to caries  Having a difficult child (*P*=.04), (ß=-2.59) was associated with caries |
| Trubey et al. (2015) | 21 | UK | Quantitative (cross-sectional survey) | 296 parents | Parent NR  Children 3 – 6 years  47.3% male, 52.7% female | NR | Frequency of parental brushing of child’s teeth in morning and evening | Time of day (evening) (Environmental context and resources) | Time of day (morning) (Environmental context and resources)  Cosmetic benefits of brushing (Beliefs about consequences)  Stable daily morning and evening routine  (Nature of behaviour) | Socioeconomic status | Over a week, parents reported brushing their children’s teeth significantly more often  in the morning (mean ± SD: 6.57 ± 1.39) than the evening (mean ± SD: 5.99 ± 2.15; Z = –3.67, p < 0.001).  Parents who lived in areas of higher socio-economic deprivation were expected to miss brushing their child’s teeth significantly more often in the morning (IRR = 3.96) and the evening (IRR = 2.07) than those parents living in less deprived areas.  Having a stable daily routine in the morning was significantly associated with a stronger morning brushing habit (r = 0.14, p < 0.05). The same significant association existed for evening routines and evening brushing habits (r = 0.15, p <  0.05). |
| Trubey et al. (2014) | 30 | UK | Qualitative (interview) | 15 parents | 2 males  Age NR  Children3-6 years old | NR | Parents’ experiences of PSB | Perception that TB frequency (i.e., once a day) is adequate compared to other parents (Social influences) | Morning and evening TB routines and habits  (Nature of behaviour)  Motivation for TB (Motivation and goals)  TB norms (Social influences)  Perception that other parents are brushing their children’s teeth more frequently (Social influences) | NR | NR |
| Vanagas et al. (2009) | 21 | Lithuania  (Kaunas) | Quantitative (Cross sectional-questionnaire) | 397 parents | 21 to 65 years (mean age, 33.1 ­_+6.94 years)  320 (80.4%) were females  Children 3-4 years | NR | Parental attitudes toward children’s oral health | NR | Parental self-efficacy to brush child’s teeth  (Beliefs about capabilities)  Parental health – oral hygiene behaviours (Social influences)  Positive parental attitudes regarding importance to brush child’s teeth  (Beliefs about consequences) | NR | There was a significant association between parents attitudes to their child’s oral health and their own oral health behaviour and an understanding of the importance of children developing oral hygiene skills (p<0.001, x^2^=29.8. |
| van Nes et al. (2018) | 31 | Netherlands | Qualitative  (focus groups and semi-structured interviews) | Two focus groups (n = 16 mothers)  Interview (n=13 mothers) | 0-5 years old | Dutch Moroccan | Knowledge, attitudes and behaviour of mothers concerning their children’s oral health. | Social norm/perception of permanent teeth being more important than primary teeth (Social influences/Beliefs about consequences)  Lack of understanding of the cause-effect relations in oral health and its translation in daily practice (Knowledge/Skills)  Difficulties supervising toothbrushing at home (e.g., child resistant) (Behaviour regulation)  Lack of self-efficacy in motivating their children to enact preventive oral self-care (Beliefs about capabilities)  Daily morning chaos and fatigue in the evening (Environmental context and resources) | Oral health knowledge broadened when having more children (Knowledge)  Openness to behavioural change (Motivation and goals)  Availability of information about oral health in schools or gatherings (Environmental context and resources/Social influences) | Migration | NR |
| Vann Jr et al. (2010) | 23 | Carolina, USA | Quantitative (structured interview) | 1,158 child/caregiver dyads | Parents 17-65 years  Children 1-59 months | White, African American, American Indians | Association between oral health literacy with knowledge, behaviour and oral health status of young children. | Low oral health literacy (Knowledge) | NR | NR | Caregivers with low oral health literacy were more likely to report poorer oral health status (OR 1.44, CI 1.02-2.05) and lower knowledge scores (OR 1.86, CI 1.41-2.45) |
| Verrips et al. (1994) | 19 | Netherlands | Quantitative (Cross-sectional – structured interview) | 476 | Children aged 5 years | Turkish, Moroccan, Dutch and Surinamese parents | Attitudes, habits and beliefs towards oral health | Poor attitudes towards importance of brushing child’s teeth (Beliefs about consequences)  Poor behavioural beliefs about efficacy and consequence of TB  (Beliefs about capabilities/Beliefs about consequences) | Behavioural training (TB skills and child behaviour management) (Skills/Behaviour regulation) | Ethnicity  Education of both parents | Parental habits, attitudes, evaluative beliefs, behavioural beliefs, perceived role of the child and that of the partner, were related commencement of TB. Large differences were observed between ethnic groups with higher risk behaviour for Turkish and Moroccan compared to Dutch and Surinamese. |
| Verrips et al. (1992) | 33 | Netherlands | Mixed (questionnaires and interviews) | 525 parents and 674 children | Parents age NR  Children5 years old  281 boys, 236 girls | Dutch, Turkish, Moroccan, Surinamese and other | Past preventive dental behaviour | Lack of knowledge regarding commencement and frequency of TB and use of F  (Knowledge) | NR | Education level of parents | Children of parents with no school education had a mean dmfs score of 4.7 higher than those of higher education |
| Virgo-Milton et al. (2016) | 24 | Australia | Semi-structured interviews | 32 mothers | Children 4-12 months  Mothers 19-42 years | 90% Australian born | View on promoting child oral health | Child temperament (Behaviour regulation)  Parental time pressures (Environmental context and resources)    Lack of knowledge (Knowledge) | Parental oral health knowledge and beliefs (Knowledge/Beliefs about consequences)  Parents’ own oral health experiences (positive or negative) (Beliefs about consequences) |  |  |
| Weinstein et al. (1999) | 10 | USA | Mixed (Structured interview) | 62 parents/caregivers | Children0-3 years old | Native American | Dental treatment experiences, concerns and caring for teeth | Greater number of children in household (Environmental context and resources)  Lack of knowledge regarding when to begin TB (Knowledge)  Expectations that child should clean own teeth at a year old (Beliefs about capabilities) | Recognition children at young age find it difficult to brush themselves (Beliefs about capabilities) | NR | NR |
| Wendt et al. (1994) | 14 | Sweden | Quantitative (structured interviews) | 629 | NR  Children1-3 years old | Sweden vs immigrants | Issues surrounding TB (frequency, regularity, use of F, problems and supervision) | Difficulty toothbrushing when child reaches 2 years old  (Behaviour regulation)  Difficulty with brushing technique (Skills) | Parental dental health information (Knowledge)  Brushing as soon as teeth erupt  (Nature of behaviour)  Training in toothbrushing techniques (Skills) | Immigration | Parents of children at different ages in non-caries group had obtained dental health information more than parents of caries- group  From non-caries group parents of children aged 1-2 years had more problems with TB than the caries group.  At age 3 parents from the non-caries group reported less problems with TB than caries group  Swedish children brushed more often than immigrants (p<0.001)  Swedish parents helped with TB >/=once a day more than immigrants (p<0.001)  The use of F was more common among Swedish parents than immigrants (TP-p<0.05 /tablets- p<0.02) |
| Wiener et al. (2009) | 11 | USA | Quantitative (Survey) | 87 | NR  Children’s mean age: 5.4  44 boys | NR | Observing parents applying TP on TB | Lack of practice of TB knowledge (recommendations)  (Skills)  Conflicting priorities (Motivation and goals) | Increased knowledge of TP and fluoride (Knowledge) | NR | NR |
| Wigen et al. (2010) | 17 | Norway | Quantitative (questionnaires) | 523 parents and their children | 60% Mothers 39% fathers and others 1% (grandparent or aunt).  Age NR  Children 5 years old | Turkey, Asia, Africa, South America, Central America and Eastern Europe. | Parents’ oral health, parents’ oral health behaviours and parents’ attitudes to oral health. | Poor attitudes regarding encouragement of TB  (Beliefs about consequences)  Parents poor oral health care habits  (Social influences) | NR | Having one or both parents of non-western origin (Ethnicity/nationality)  Low education level | Children were more likely to have caries at five years old if they had one or both parents of non-western origin (OR = 4.8; both OR = 3.0; one parent OR = 2.1), parents with low education levels, lenient parental attitudes towards child’s TB (OR = 2.8), and having parents’ who brushed own teeth less than twice a day (OR = 2.2) |
| Wilson et al. (2014) | 21 | Northern Plains reservation, Colorado, America | Quantitative (Survey) | 147 parents | Parents 15-54 years  Children 0-7 years old | American Indian | Association between sociodemographic variables and  oral health knowledge and behaviours | Lack of knowledge that young children need assistance brushing teeth, and when children can brush teeth by themselves) (Knowledge) | Greater oral health knowledge (Knowledge) |  | Behaviour (including assisting child with TB) was significantly associated with knowledge scores (p=0.016) |
| Wilson et al. (2017) | **27** | USA | Quantitative (Cross-sectional) | 100 mother–child dyads | Mean age of participating children was 4.0 + 1.1 years and 46.5% were female. Mean age of participating mothers was 31.4 + 6.6 years, | Latino | Individual (i.e., knowledge, behaviour, and  oral health outcomes) and cultural level factors (acculturation)  and their importance/inter-relationships with oral health | Perception caries is not a serious condition not child susceptible to caries (Beliefs about consequences) | Knowledge (Knowledge)  Perceptions of benefits to adhering to recommended oral health behaviours and child being susceptible to caries (Beliefs about consequences)  Confidence in ability to manage child’s oral health (Beliefs in capabilities) | Mother’s level of education  Duration in household | NR |
| Wong et al. (2005) | 18 | New York, USA | Qualitative (interview) | 22 parents from 20 interviews  24 children with extensive caries | Children under 12 years old | Chinese | Beliefs and perspectives  related to caries, oral health habits, and dental treatment | Belief children should be brushing own teeth  (Beliefs about capabilities)  Lack of access to dental provisions (Environmental context and resources) | Noticing signs of dental caries in child (Knowledge)  Fears about effects of general anaesthetic (Emotion) |  | NR |
| Wyne et al. (1997) | 12 | Australia | Quantitative (survey) | 160 parents and their children | NR  88 boys | NR | TB practices of children | NR | Advice on TB practices in early childhood (Knowledge) | NR | NR |
| Zeedyk et al. (2005) | 34 | UK | Observational study | 18 families | NR  Children 2.5 years old (31-33 months) | NR | Assess TB behaviour of parents and children | Lack of confidence in parents (Beliefs about capabilities)  Child’s lack of concentration and cooperation/Unwillingness of child to take a turn in TB (Behaviour regulation) | NR | NR | Parents’ perceptions of a tooth-brushing  session was not associated with the behaviours demonstrated during TB (r = 0.02 total time brush in child’s mouth, and r=0.19 length of time parents in control of brush) |

Note: B – Barriers; dmft – decayed, missing, filled teeth; F – Facilitators; FTP – Fluoride toothpaste; NR – Not reported; PSB – Parental Supervised Toothbrushing; SES – Socioeconomic status; TB – Toothbrushing, TP – Toothpaste.
