# Supplementary material for: Facilitators and barriers to home-based toothbrushing practices by parents of young children to reduce tooth decay: a systematic review
Source: Clin Oral Investig. 2021 Mar 20;25(6):3383–93. doi: 10.1007/s00784-021-03890-z (PMC8137613; doi:10.1007/s00784-021-03890-z)
Supplement: Supplementary file 3 — (DOCX 81 kb) [file 784_2021_3890_MOESM3_ESM.docx]

**Supplementary materials 2**

References for studies included in systematic review

1. Adair PM, Pine CM, Burnside G, et al. Familial and cultural perceptions and beliefs of oral hygiene and dietary practices among ethnically and socio-economicall diverse groups. *Community Dental Health.* 2004;21(1 Suppl):102-111.

2. Adiatman M, Zhafarina AR, Rahardjo A, Badruddin IA, Prabawanti C. The correlation between mothers' behaviors of maintaining their children's oral hygiene and early childhood caries (based on the theory of planned behavior). *Journal of International Dental and Medical Research.* 2017;10(Specialissue):619-627.

3. Akpabio A, Klausner CP, Inglehart MR. Mothers'/guardians' knowledge about promoting children's oral health. *J Dent Hyg.* 2008;82(1):12.

4. Amin MS, Harrison RL. Understanding Parents' Oral Health Behaviors for Their Young Children. *Qualitative Health Research.* 2009;19(1):116-127.

5. Ashkanani F, Al-Sane M. Knowledge, attitudes and practices of caregivers in relation to oral health of preschool children. *Medical Principles and Practice.* 2013;22(2):167-172.

6. Ayoub S. Influence of maternal psychosocial factors on child's oral health behavior. *Dissertation Abstracts International: Section B: The Sciences and Engineering.* 2017;78(3-B(E)):No Pagination Specified.

7. Baginska J, Rodakowska E. *Knowledge and practice of caries prevention in mothers from Bialystok, Poland.* International Journal of Collaborative Research on Internal Medicine and Public Health. 4 (5) (pp 431-441), 2012. Date of Publication: May 2012.; 2012.

8. Begzati A, Bytyci A, Meqa K, Latifi-Xhemajli B, Berisha M. Mothers' behaviours and knowledge related to caries experience of their children. *Oral Health & Preventive Dentistry.* 2014;12(2):133-140.

9. Bennadi D, Kshetrimayum N, Sibyl S, Reddy CVK. *Toothpaste utilization profiles among preschool children.* Journal of Clinical and Diagnostic Research. 8 (3) (pp 212-215), 2014. Date of Publication: 15 Mar 2014.; 2014.

10. Blinkhorn AS. Influence of social norms on toothbrushing behavior of preschool-children. *Community dentistry and oral epidemiology.* 1978;6(5):222-226.

11. Blinkhorn AS, Wainwright-Stringer YM, Holloway PJ. Dental health knowledge and attitudes of regularly attending mothers of high-risk, pre-school children. *International dental journal.* 2001;51(6):435-438.

12. Boustedt K, Dahlgren J, Twetman S, Roswall J. Tooth brushing habits and prevalence of early childhood caries: a prospective cohort study. *European Archives of Paediatric Dentistry.* 2019.

13. Bozorgmehr E, Hajizamani A, Malek Mohammadi T. *Oral health behavior of parents as a predictor of oral health status of their children.* ISRN Dentistry. 2013 , 2013. Article Number: 741783. Date of Publication: 2013.; 2013.

14. Broder H, Reisine S, Johnson R. Role of african-american fathers in child-rearing and oral health practices in an inner city environment--a brief communication. *Journal of Public Health Dentistry.* 2006;66(2):138-143.

15. Carvalho JC, Silva EF, Vieira EO, Pollaris A, Guillet A, Mestrinho HD. Oral Health Determinants and Caries Outcome among Non-Privileged Children. *Caries Research.* 2014;48(6):515-523.

16. Chen CC, Chiou SJ, Ting CC, et al. Immigrant-native differences in caries-related knowledge, attitude, and oral health behaviors: a cross-sectional study in Taiwan. *Bmc Oral Health.* 2014;14.

17. Chhabra N, Chhabra A. Parental knowledge, attitudes and cultural beliefs regarding oral health and dental care of preschool children in an Indian population: a quantitative study. *Eur Arch Paediatr Dent.* 2012;13(2):76-82.

18. Collett BR, Huebner CE, Seminario AL, Wallace E, Gray KE, Speltz ML. Observed child and parent toothbrushing behaviors and child oral health. *International Journal of Paediatric Dentistry.* 2016;26(3):184-192.

19. Daly J, Levy S, Xu Y, Jackson R, Eckert G, Levy B. Factors Associated With Parents’ Perceptions of Their Infants’ Oral Health Care. *Journal of Primary Care & Community Health.* 2016;7(3):180-187.

20. Danila I, Samoila A. Oral health behavior, knowledge, and attitudes of children, mothers, and schoolteachers in Romania in 1993. *Acta Odontologica Scandinavica.* 1995;53(6):363-368.

21. Davidovich E, Kooby E, Shapira J, Ram D. Oral hygiene habits, dental home, and toothbrushing among immigrant and native low socioeconomic class populations. *Journal of Clinical Pediatric Dentistry.* 2013;37(4):341-344.

22. Duijster D, de Jong-Lenters M, de Ruiter C, Thijssen J, van Loveren C, Verrips E. Parental and family-related influences on dental caries in children of Dutch, Moroccan and Turkish origin. *Community Dentistry and Oral Epidemiology.* 2015;43(2):152-162.

23. Duijster D, de Jong-Lenters M, Verrips E, van Loveren C. Establishing oral health promoting behaviours in children - parents' views on barriers, facilitators and professional support: a qualitative study. *Bmc Oral Health.* 2015;15:13.

24. Duijster D, Verrips GHW, Van Loveren C. The role of family functioning in childhood dental caries. *Community Dentistry and Oral Epidemiology.* 2014;42(3):193-205.

25. Ekman A, Holm AK, Schelin B, Gustafsson L. Dental health and parental attitudes in Finnish immigrant preschoolchildren in the north of Sweden. *Community Dent Oral Epidemiol.* 1981;9(5):224-229.

26. Finlayson TL, Siefert K, Ismail AI, Delva J, Sohn W. Reliability and validity of brief measures of oral health-related knowledge, fatalism, and self-efficacy in mothers of African American children. *Pediatric Dentistry.* 2005;27(5):422-428.

27. Hamilton K, Cornish S, Kirkpatrick A, Kroon J, Schwarzer R. Parental supervision for their children's toothbrushing: Mediating effects of planning, self-efficacy, and action control. *British Journal of Health Psychology.* 2018;23(2):387-406.

28. Huebner CE, Riedy CA. Behavioral determinants of brushing young children's teeth: implications for anticipatory guidance. *Pediatric Dentistry.* 2010;32(1):48-55.

29. Janson S, Fakhouri H. Dental health in suburban Jordanian preschool children. *Swedish Dental Journal.* 1993;17(3):123-127.

30. Lenčová E, Dušková J. Oral health attitudes and caries-preventive behaviour of Czech parents of preschool children. *2013.* 2013;42(2).

31. Marshman Z, Ahern SM, McEachan RRC, Rogers HJ, Gray-Burrows KA, Day PF. Parents’ experiences of toothbrushing with children: A qualitative study. *JDR Clinical and Translational Research.* 2016;1(2):122-130.

32. Mofidi M, Zeldin LP, Rozier R. Oral health of early head start children: A qualitative study of staff, parents, and pregnant women. *American Journal of Public Health.* 2009;99(2):245-251.

33. Nagarajappa R, Kakatkar G, Sharda AJ, Asawa K, Ramesh G, Sandesh N. Infant oral health: Knowledge, attitude and practices of parents in Udaipur, India. *Dental research journal.* 2013;10(5):659-665.

34. Naidu RS, Davis L. Parents' views on factors influencing the dental health of Trinidadian pre-school children. *Community Dental Health.* 2008;25(1):44-49.

35. Naidu R, Nunn J, Forde M. Oral healthcare of preschool children in Trinidad: a qualitative study of parents and caregivers. *BMC Oral Health.* 2012;12.

36. Narksawat K, Boonthum A, Tonmukayakul U. Roles of parents in preventing dental caries in the primary dentition among preschool children in Thailand. *Asia-Pacific Journal of Public Health.* 2011;23(2):209-216.

37. Paunio P, Rautava P, Helenius H, Sillanpaa M. Children's poor toothbrushing behavior and mothers' assessment of dental health education at well-baby clinics. *Acta Odontologica Scandinavica.* 1994;52(1):36-42.

38. Petersen PE. Oral health behavior of 6-year-old Danish children. *Acta Odontologica Scandinavica.* 1992;50(1):57-64.

39. Petersen PE, Esheng Z. Dental caries and oral health behaviour situation of children, mothers and schoolteachers in Wuhan, People's Republic of China. *International Dental Journal.* 1998;48(3):210-216.

40. Pine CM, Adair PM, Nicoll AD, et al. International comparisons of health inequalities in childhood dental caries. *Community dental health.* 2004;21(1 Suppl):121-130.

41. Pisarnturakit PP, Shaw BR, Tanasukarn C, Vatanasomboon P. Validity and reliability of the early childhood caries perceptions scale (ECCPS) to assess health beliefs related to early childhood caries prevention among primary caregivers of children under 5 years of age. *Southeast Asian Journal of Tropical Medicine and Public Health.* 2012;43(5):1280-1291.

42. Prowse S, Schroth RJ, Wilson A, et al. Diversity considerations for promoting early childhood oral health: a pilot study. *International journal of dentistry.* 2014;2014:175084.

43. Rahbari M, Gold J. Knowledge and behaviors regarding early childhood caries among low-income women in Florida: a pilot study. *Journal of Dental Hygiene.* 2015;89(2):132-138.

44. Reisine S, Ajrouch KJ, Sohn W, Lim S, Ismail A. : Brief communications. *Journal of Public Health Dentistry.* 2009;69(3):197-200.

45. Senesombath S, Nakornchai S, Banditsing P, Lexomboon D. Early childhood caries and related factors in Vientiane, Lao PDR. *Southeast Asian J Trop Med Public Health.* 2010;41(3):717-725.

46. Spitz AS, Weber-Gasparoni K, Kanellis MJ, Qian F. Child temperament and risk factors for early childhood caries. *Journal of dentistry for children (Chicago, Ill).* 2006;73(2):98-104.

47. Sujlana A, Pannu PK. Family related factors associated with caries prevalence in the primary dentition of five-year-old children. *Journal of Indian Society of Pedodontics and Preventive Dentistry.* 2015;33(2):83-87.

48. Sun XY, Bernabe E, Liu XN, Gallagher JE, Zheng SG. Early life factors and dental caries in 5-year-old children in China. *Journal of Dentistry.* 2017;64:73-79.

49. Sutthavong S, Taebanpakul S, Kuruchitkosol C, et al. Oral health status, dental caries risk factors of the children of public kindergarten and schools in Phranakornsriayudhya, Thailand. *J Med Assoc Thai.* 2010;93 Suppl 6:S71-78.

50. Szatko F, Wierzbicka M, Dybizbanska E, Struzycka I, Iwanicka-Frankowska E. Oral health of Polish three-year-olds and mothers' oral health-related knowledge. *Community dental health.* 2004;21(2):175-180.

51. Tiberia MJ, Milnes AR, Feigal RJ, et al. Risk factors for early childhood caries in Canadian preschool children seeking care. *Pediatric Dentistry.* 2007;29(3):201-208.

52. Trubey RJ, Moore SC, Chestnutt IG. Children's Toothbrushing Frequency: The Influence of Parents' Rationale for Brushing, Habits and Family Routines. *Caries Research.* 2015;49(2):157-164.

53. Trubey RJ, Moore SC, Chestnutt IG. Parents' reasons for brushing or not brushing their child's teeth: a qualitative study. *International Journal of Paediatric Dentistry.* 2014;24(2):104-112.

54. Vanagas G, Milasauskiene Z, Grabauskas V, Mickeviciene A. Associations between parental skills and their attitudes toward importance to develop good oral hygiene skills in their children. *Medicina (Kaunas, Lithuania).* 2009;45(9):718-723.

55. van Nes KA, Veerkamp JSJ, Reis R. Barriers and opportunities to oral health in Dutch-Moroccan children in the Netherlands: a narrative report. *European Archives of Paediatric Dentistry.* 2018;19(5):353-364.

56. Vann Jr WF, Lee JY, Baker D, Divaris K. Oral health literacy among female caregivers: Impact on oral health outcomes in early childhood. *Journal of Dental Research.* 2010;89(12):1395-1400.

57. Verrips GH, Kalsbeek H, Van Woerkum CM, Koelen M, Kok-Weimar TL. Correlates of toothbrushing in preschool children by their parents in four ethnic groups in The Netherlands. *Community Dental Health.* 1994;11(4):233-239.

58. Verrips GH, Frencken JE, Kalsbeek H, ter Horst G, Filedt Kok-Weimar TL. Risk indicators and potential risk factors for caries in 5-year-olds of different ethnic groups in Amsterdam. *Community Dent Oral Epidemiol.* 1992;20(5):256-260.

59. Virgo-Milton M, Boak R, Hoare A, et al. An exploration of the views of Australian mothers on promoting child oral health. *Australian Dental Journal.* 2016;61(1):84-92.

60. Weinstein P, Troyer R, Jacobi D, Moccasin M. Dental experiences and parenting practices of Native American mothers and caretakers: what we can learn for the prevention of baby bottle tooth decay. *J Dent Child.* 1999;66(2):120-126, 185.

61. Wendt LK, Hallonsten AL, Koch G, Birkhed D. Oral hygiene in relation to caries development and immigrant status in infants and toddlers. *Scand J Dent Res.* 1994;102(5):269-273.

62. Wiener RC, Crout RJ, Wiener MA. Toothpaste use by children, oral hygiene, and nutritional education: an assessment of parental performance. *J Dent Hyg.* 2009;83(3):141-145.

63. Wigen TI, Wang NJ. Caries and background factors in Norwegian and immigrant 5-year-old children. *Community Dent Oral Epidemiol.* 2010;38(1):19-28.

64. Wilson A, Brega AG, Batliner TS, et al. Assessment of parental oral health knowledge and behaviors among American Indians of a Northern Plains tribe. *Journal of Public Health Dentistry.* 2014;74(2):159-167.

65. Wilson AR, Mulvahill MJ, Tiwari T. The impact of Maternal self-efficacy and Oral health Beliefs on early childhood caries in latino children. *Frontiers in Public Health.* 2017;5:10.

66. Wong D, Perez-Spiess S, Julliard K. Attitudes of Chinese parents toward the oral health of their children with caries: a qualitative study. *Pediatric dentistry.* 2005;27(6):505-512.

67. Wyne AH, Spencer AJ, Szuster FS. Toothbrushing practices of 2-3-year-old children and their age at first dental visit: a survey in Adelaide, South Australia. *International journal of paediatric dentistry / the British Paedodontic Society [and] the International Association of Dentistry for Children.* 1997;7(4):263-264.

68. Zeedyk MS, Longbottom C, Pitts NB. Tooth-brushing practices of parents and toddlers: a study of home-based videotaped sessions. *Caries Research.* 2005;39(1):27-33.
